# PDGF gene expression and p53 alterations contribute to the biology of diffuse astrocytic gliomas

**DOI:** 10.1038/s41525-023-00351-2

**Published:** 2023-02-25

**Authors:** Mehul Kumar, Mathieu Meode, Michael Blough, Gregory Cairncross, Pinaki Bose

**Affiliations:** 1 Department of Biochemistry and Molecular Biology, University of Calgary, Calgary, AB Canada; 2 Charbonneau Cancer Institute, University of Calgary, Calgary, AB Canada; 3 Department of Clinical Neurosciences, University of Calgary, Calgary, AB Canada; 4 Hotchkiss Brain Institute, University of Calgary, Calgary, AB Canada; 5 Department of Oncology, University of Calgary, Calgary, AB Canada

**Keywords:** Oncogenesis, Prognostic markers

## Abstract

Diffuse, histologically lower grade astrocytomas of adults (LGAs) are classified based on the mutational status of the isocitrate dehydrogenase (*IDH*) genes. While wild-type (WT) LGAs often evolve quickly to glioblastoma (GBM), mutant tumors typically follow an indolent course. To find possible effectors of these different behaviors, we compared their respective transcriptomes. Unlike mutant LGAs, platelet-derived growth factor (PDGF) signaling was significantly enriched in WT tumors, and *PDGFA* was the top overexpressed gene in the pathway. Moreover, methylation of the *PDGFA* and *PDGFD* promoters emerged as a possible mechanism for their low expression in mutant tumors. Copy number gain of chromosome 7 co-occurred with high expression of *PDGFA* in WT cases, and high expression of *PDGFA* was associated with aneuploidy, extracellular matrix (ECM)-related immunosuppressive features and poor prognosis. We also noted that high *PDGFA* expression in WT cases occurred irrespective of tumor grade and that multiple mechanisms of p53 pathway inactivation accompanied progression to GBM in *PDGFA*-overexpressing tumors. Conversely, *TP53* point mutations were an early and constant feature of mutant LGAs. Our results suggest that members of the PDGF gene family, in concert with different p53 pathway alterations, underlie LGA behaviors.

## Introduction

Diffuse fibrillary astrocytomas of adults (WHO grades II and III), collectively termed lower grade astrocytomas (LGAs), are a group of deadly brain cancers with unknown etiology^1^. A significant insight into their biology and variable clinical behavior began to emerge when isocitrate dehydrogenase 1 and 2 (*IDH1/2*) mutations were discovered in 10% of glioblastomas (GBMs) and subsequently in a large proportion of LGAs^2^. Further characterization revealed that *IDH* wild-type (WT) LGAs usually arose in older adults and tended to evolve rapidly to GBM, whereas *IDH* mutant LGAs occurred in younger adults, grew slowly, and only sometimes evolved into a GBM-like high-grade cancer^2–4^. Despite their similar histology, *IDH* WT and mutant LGAs had different natural histories. It should be noted that a clinical nuance of the *IDH* WT LGAs, namely their predictable evolution to higher grades, has been muted now that many of them have been re-designated, GBMs. Soon thereafter, molecular and biochemical features that distinguish *IDH* WT LGAs from mutant tumors were identified, including amplification of chromosome 7, deletion of chromosome 10, and mutations of the *TERT* gene promoter in *IDH* WT cases *versus* loss of the alpha-thalassemia x-linked (*ATRX*) gene, point mutations of the *TP53* gene, and production of the oncometabolite, 2-hydroxyglutarate (2-HG) in *IDH* mutant LGAs^5,6^. How these *IDH*-dependent alterations influence the behavior of LGAs is unknown^7^.

To address this issue, we analyzed whole transcriptome data from non-1p/19q co-deleted LGAs in The Cancer Genome Atlas (TCGA)^5^ and found interesting differences between *IDH* WT and mutant LGAs with respect to PDGF signaling, especially the association of *PDGFA* and *PDGFD* gene expression with promoter methylation and copy number variations (CNVs) in LGAs. We also found an association between overexpression of *PDGFA* and *PDGFD* and aneuploidy, markers of immuno-suppression, and poor survival outcome. Furthermore, we noted that the progression of WT LGAs to GBM was associated with inactivation of multiple elements of the p53 pathway and differed from mutant LGAs in this respect, where p53 point mutations were an early and constant finding. Our results point to the cooption of aberrant PDGF and p53 signaling in the progression of *IDH* WT astrocytomas.

## Results

### PDGF pathway enrichment and high expression of PDGFA were observed in IDH WT LGAs

**Fig. 1:**
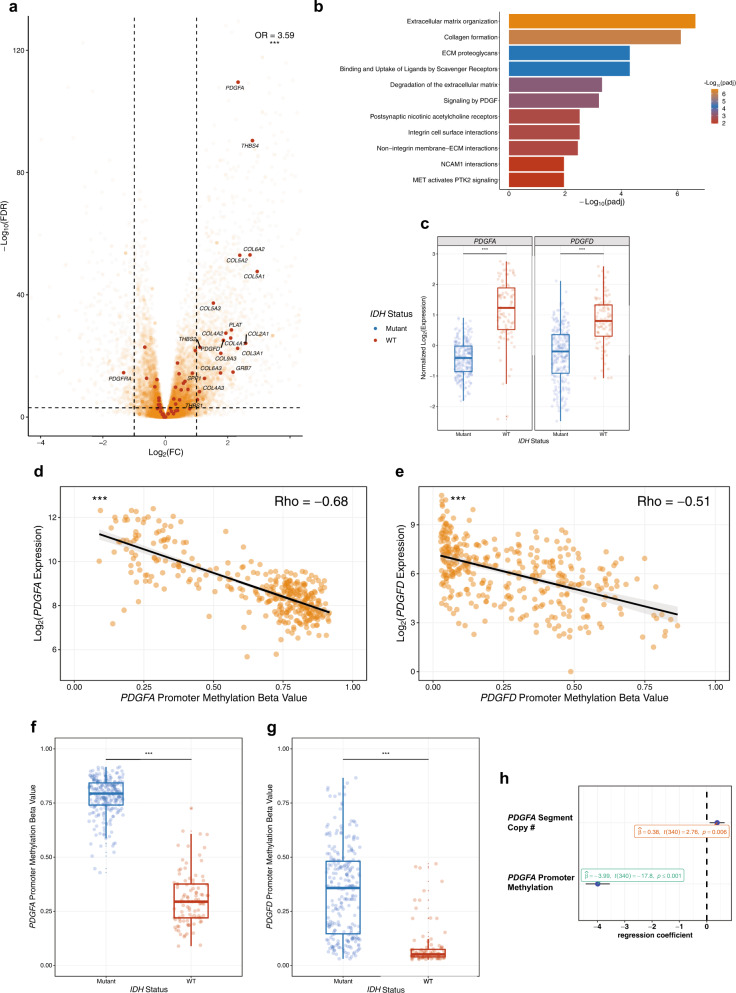
*PDGFA* and *PDGFD* expression are dysregulated in *IDH* WT LGAs. **a** Volcano plot showing fold changes for genes differentially expressed between *IDH* WT and *IDH* mutant LGAs. PDGF pathway members are enriched in the overexpressed genes (maroon dots). Positive Log_2_(FC) indicates upregulation in *IDH* WT LGAs. **b** Reactome pathway analysis of genes overexpressed in *IDH* WT LGAs reveals the enrichment of ECM-associated genes and the PDGF signaling pathway. **c** Unbiased tSNE visualization with gene expression values of PDGF pathway genes separates LGAs by *IDH* mutation status. *PDGFA* and *PDGFD* gene expression are significantly elevated in *IDH* WT LGAs, relative to *IDH* mutant LGAs. **d**, **e** Scatterplots showing the negative correlation of promoter methylation with *PDGFA* and *PDGFD* expression in LGAs. Spearman’s Rho values are reported as a measure of effect size from the Spearman’s Rank-Order Correlation test. **f**, **g** Box plots showing that promoter methylation of *PDGFA* and *PDGFD* are elevated in *IDH* mutant relative to WT LGAs. **h** Multivariate linear model showing the independent association of *PDGFA* expression with *PDGFA* promoter methylation and copy number of the segment containing *PDGFA* on chromosome 7. OR Odds Ratio. (***P < 0.001); in Box plots, the lower bound, center line and upper bound correspond to the first, second and third quartiles, respectively, and whiskers correspond to the maximum and minimum data values.

To explore putative mechanisms underlying the differences in behavior of *IDH* WT and mutant LGAs, we performed differential expression analysis (DEA) on all LGAs from a filtered TCGA dataset (*n* = 347) stratified by *IDH1*/*2*-mutation status. In this analysis we found 2,175 overexpressed and 517 downregulated genes in *IDH* WT LGAs (*n* = 94) *versus IDH* mutant cases (*n* = 250; adjusted *P* value = 0.001, log_2_(fold change) = 1; Fig. 1a). To assess the functional significance of differentially expressed genes, we performed canonical Reactome pathway analysis: enriched pathways in *IDH* WT tumors included ECM deregulation (adjusted *P* = 2.3 * 10^−7^), collagen biosynthesis (adjusted *P* = 7.6 * 10^−7^), and PDGF signaling (adjusted *P* = 6.3 * 10^−4^) (Fig. 1b). Enrichment of ECM pathways^8^ and prominence of the PDGF pathway were of interest because of the invasive nature of LGAs and because overexpression of *PDGFA* has been implicated in the pathogenesis *IDH* WT GBM^9^ and exposure to PDGFA is able to transform p53 null neural progenitor cells^10^.

The most differentially expressed gene in the PDGF family^11^ was *PDGFA* (Fig. 1a, b). *PDGFA* (adjusted *P* = 2.7 * ^−110^, log_2_(fold change) = 2.33), like *PDGFD* (adjusted *P* = 8.3 * 10^−26^, log_2_(fold change) = 1.86), was significantly upregulated in *IDH* WT LGAs compared to mutant LGAs (Fig. 1a, d), where in contrast to the PDGFA/PDGFD ligands, the receptor *PDGFRA*^12^ was overexpressed (Fig. 1a). Other members of the PDGF pathway such as *PDGFB, PDGFC* and *PDGFRB* were not differentially expressed in *IDH* WT *versus* mutant LGAs.

We then explored mechanisms underlying the differential expression of *PDGFA* and *PDGFD* in LGAs. Aware that hypermethylation is a feature of *IDH* mutant tumors^13^, we asked whether promoter methylation was associated with *PDGFA/PDGFD* expression and documented a strong negative correlation between expression and methylation of both genes across all LGAs (*PDGFA*: *P* < 2.2 * 10^−16^, Spearman’s Rho = −0.68, *n* = 347, Fig. 1d) (*PDGFD*: *P* < 2.2 * 10^−16^, Spearman’s Rho = −0.51, *n* = 347, Fig. 1e). Significantly lower amounts of *PDGFA* (Fig. 1f) and *PDGFD* (Fig. 1g) promoter methylation was observed in *IDH* WT LGAs (*n* = 94) compared to mutant cases (*n* = 250) (univariate comparisons for both genes: *P* = 2.2 * 10^−16^). The negative correlation between expression and promoter methylation persisted when *IDH* WT and mutant LGAs were analyzed separately (Supplementary Fig. 1a–d), indicating that promoter methylation may be an important regulatory mechanism of *PDGFA*/*PDGFD* expression in both LGA subtypes.

Next, we investigated the correlation between gene expression and copy number to assess whether chromosome 7 (containing the *PDGFA* locus) and 11 (containing the *PDGFD* locus) gains were associated with differential expression of these genes. As previously reported^14^, we found that a significantly higher proportion of WT LGAs displayed amplification of the portion of chromosome 7 containing *PDGFA* (hg19: Chr 7: 536897 base pairs (bp) to 559481 bp) than mutant LGAs (*P* = 2.2 * 10^−16^, *n* = 343, Supplementary Fig. S2a. In contrast to *PDGFA*, segmental amplification of *PDGFD* (hg19: Chr 11: 103777914 bp to 104035027 bp) was not a feature of WT LGAs. Fifty-six percent of WT tumors had *PDGFA* amplification (Supplementary Fig. S2a) but only 1% displayed *PDGFD* amplification (Supplementary Fig. S3A). Indeed, for *PDGFD*, the frequency of amplification was higher in mutant tumors (*P* = 0.036, *n* = 345, Supplementary Fig. S2b), although the percentage of mutant tumors with amplification of *PDGFD* was relatively low at 7% (Supplementary Fig. S2b).

Furthermore, the absolute copy number of the PDGFA-containing segment on chromosome 7 significantly correlated with *PDGFA* expression in *IDH* WT LGAs (*P* = 0.0022, Spearman’s Rho = +0.31, *n* = 93, Supplementary Fig. S2c), but not in mutant cases (*P* = 0.97, Spearman’s Rho = +0.00, *n* = 247, Supplementary Fig. S2d). In a multivariate linear regression model, both *PDGFA* promoter methylation (*P* < 2.2 * 10^−16^, t-value = −17.804) and the copy number of the *PDGFA*-containing segment (*P* = 0.0062, t-value = 2.755) were significantly associated with its expression in all LGAs (*n* = 347) (Fig. 1h). These results reveal a previously unrecognized mechanism by which PDGF signaling can be regulated in LGAs. In *IDH* WT LGAs, absence of promoter methylation of *PDGFA* and *PDGFD* and amplification of chromosome 7 contribute to higher gene expression, whereas in *IDH* mutant LGAs, hypermethylation of the *PDGFA* and *PDGFD* promoters and absence of chromosome 7 amplification are significantly associated with the decreased expression of *PDGFA* and *PDGFD*.

### Gene expression and promoter methylation of PDGFA and PDGFD, and amplification of PDGFA, were significantly associated with prognosis among LGA patients

**Fig. 2:**
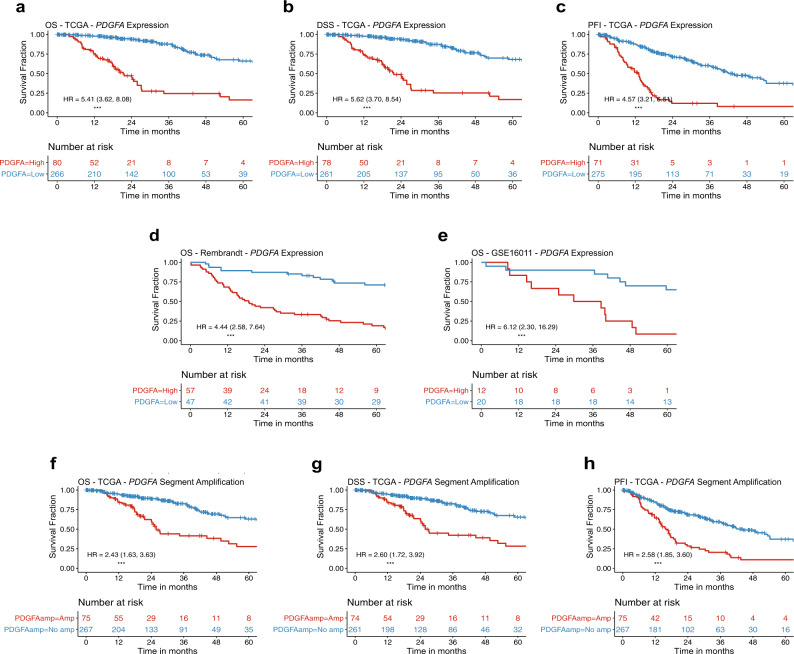
*PDGFA* expression and amplification status are associated with worse prognosis in LGAs. **a–c** KM survival curves for OS, DSS and PFI showing the separation of TCGA LGA patients into high-risk groups based on *PDGFA* expression. **d**, **e** KM survival curves validating the association between *PDGFA* expression of the tumor and overall survival of the patient in LGA samples from the REMBRANDT and GSE16011 datasets. **f–h** KM survival curves for OS, DSS and PFI showing the separation of TCGA LGA patients into risk groups based on whether the chromosomal segment containing the *PDGFA* locus is amplified or not. Hazard ratios (HR) and their respective 95% confidence intervals from univariate Cox proportional hazards analysis of the dichotomized expression groups are shown for each KM curve. (***P < 0.001).

Cox proportional hazards (PH) analysis was performed, and Kaplan–Meier (KM) curves were generated to assess whether gene expression and/or promoter methylation of *PDGFA* and *PDGFD* were prognostic factors in LGAs. Higher *PDGFA* expression was associated with significantly worse overall survival (OS) (*P* = 8 * 10^−13^, HR = 1.67, 95% C.I. [1.45, 1.93], *n* = 347, disease specific survival (DSS) (*P* = 2.8 * 10^−12^, HR = 1.69, 95% C.I. [1.46, 1.95], *n* = 347, and progression-free interval (PFI) (*P* = 8.9 * 10^−14^, HR = 1.00, 95% C.I. [1.00, 1.00], *n* = 347, (Fig. 2a–c). These results were confirmed in two additional datasets: REpository for Molecular BRAin Neoplasia DaTa (REMBRANDT)^15^ (*P* = 5.9 * 10^−6^, HR = 2.22, 95% C.I. [1.57, 3.14], *n* = 109 (Fig. 2d) and GSE16011^16^ (*P* = 0.0031, HR = 1.67, 95% C.I. [1.19, 2.35], *n* = 32 (Fig. 2e), suggesting that *PDGFA* expression is a prognostic biomarker in LGA. Similar prognostic associations were observed for *PDGFD* expression (Supplementary Fig. S3a–e). Lower *PDGFA* and *PDGFD* promoter methylation (Supplementary Fig. S4a–f) and amplification of the chromosome segment containing *PDGFA* (Fig. 2f–h) were also associated with shorter OS, DSS, and PFI. These data suggest that mechanisms regulating the expression of *PDGFA* and *PDGFD* affect the clinical outcomes and biology of patients with LGAs.

### PDGFA and PDGFD gene expression and IDH WT status were associated with aneuploidy and markers of immuno-suppression

**Fig. 3:**
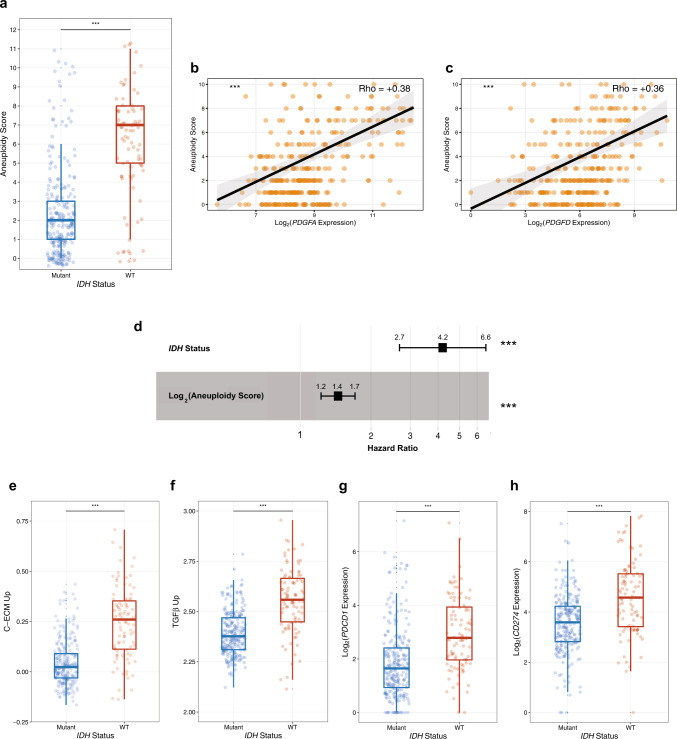
*PDGFA* and *PDGFD* expression are associated with markers of immuno-suppression in LGA patients. **a** Box plots depicting the quantification of aneuploidy scores (AS) in *IDH* WT and mutant LGAs. **b**, **c** Scatterplots showing the correlations between AS and expression of *PDGFA* and *PDGFD*, respectively; Spearman’s Rho values are reported as a measure of effect size from the Spearman’s Rank-Order Correlation test. **d** Forest plot derived from a multivariate Cox proportional hazards regression model for OS using *IDH* mutation status and Log_2_(AS + 1) as covariates. Hazard Ratio (HR) and the respective 95% C.I. are shown above the points; a HR > 1 indicates that *IDH* WT status and high AS are associated with worse OS. **e–h** Box plots comparing the distributions of ssGSEA scores for C-ECM upregulated genes, TGF-β upregulated target genes, *PDCD1* (PD-1) expression, and *CD274* (PD-L1) expression, respectively, between *IDH* WT and mutant LGAs. (***P < 0.001). In Box plots, the lower bound, center line and upper bound correspond to the first, second and third quartiles, respectively, and whiskers correspond to the maximum and minimum data values.

Given the worse prognosis of *IDH* WT LGAs patients that overexpress *PDGFA*, we assessed additional biological features of these tumors that might explain their propensity for more aggressive behavior. Having recently reported that in vitro exposure to PDGFA leads to chromosomal instability in neural progenitor cells^10^, we assessed aneuploidy in LGAs in relation to *IDH* mutational status and *PDGFA* expression. We observed that *IDH* WT LGAs were significantly more aneuploid than their *IDH* mutant counterparts (*P* = 2.2 * 10^−16^, *n* = 341, Fig. 3a). We further observed that aneuploidy was a distinguishing feature of LGAs that expressed high levels of *PDGFA* and *PDGFD*. Aneuploidy score (AS)^17^ was significantly associated with expression of *PDGFA* (*P* = 6.8 * 10^−13^, Spearman’s Rho = +0.38, *n* = 338) and *PDGFD* (*P* = 6.9 * 10^−12^, Spearman’s Rho = +0.36, *n* = 338) in univariate analysis (Fig. 3b, c, respectively). Furthermore, univariate Cox PH analyses revealed that higher AS was associated with worse OS (*P* = 1.1 * 10^−11^, HR = 1.76, 95% C.I. [1.50, 2.07], *n* = 341, DSS (*P* = 2.7 * 10^−11^, HR = 1.78, 95% C.I. [1.50, 2.10], *n* = 334, and PFI (*P* = 1.2 * 10^−9^, HR = 1.50, 95% C.I. [1.32, 1.71], *n* = 341) (Supplementary Fig. S5a–c). In multivariate Cox PH analysis, both AS (*P* = 9.5 * 10^−5^, HR = 1.42, 95% C.I. [1.19, 1.70]) and *IDH* status (*P* = 3.2 * 10^−10^, HR = 4.20, 95% C.I. [2.69, 6.57]) remained independent predictors of overall survival in LGAs (*n* = 347, Fig. 3d). These analyses indicate that the presence of aneuploidy has prognostic value independent of *IDH* status in LGAs, and that aneuploidy is associated with high expression of the *PDGFA* and *PDGFD* genes.

We then explored the potential of LGAs for immune evasion, a hallmark of poor prognosis across cancers^18,19^. As observed in our pathway enrichment analysis, ECM genes were upregulated in *IDH* WT LGAs (Fig. 1b). This is an intriguing observation given that we have previously reported that ECM dysregulation is an effector of TGF-β-induced immuno-suppression in the tumor microenvironment^20^. To explore this result further, we investigated immune suppression in LGAs with respect to their *IDH* mutational status and documented that WT LGAs had significantly higher expression of cancer-associated ECM (C-ECM) genes (*P* = 2.2 * 10^−16^, *n* = 344, Fig. 3e) and TGF-β upregulated target genes (*P* = 2.2 * 10^−16^, *n* = 344, Fig. 3f). Furthermore, in all LGAs (*n* = 347), the expression of *PDGFA* and *PDGFD* were positively correlated with both features (Supplementary Fig. S6a–d). The expression of immunosuppressive checkpoint genes such as *PDCD1* (encodes PD-1) (*P* = 2.3 * 10^−11^) and *CD274* (encodes PD-L1) (*P* = 1.1 * 10^−9^) were also increased in *IDH* WT LGAs (Fig. 3g, h, *n* = 344), suggesting that WT tumors may be able to suppress the local immune response to enhance their aggressiveness.

### Sustained overexpression of PDGFA and progressive inactivation of the p53 pathway characterized the evolution of IDH WT LGAs

**Fig. 4:**
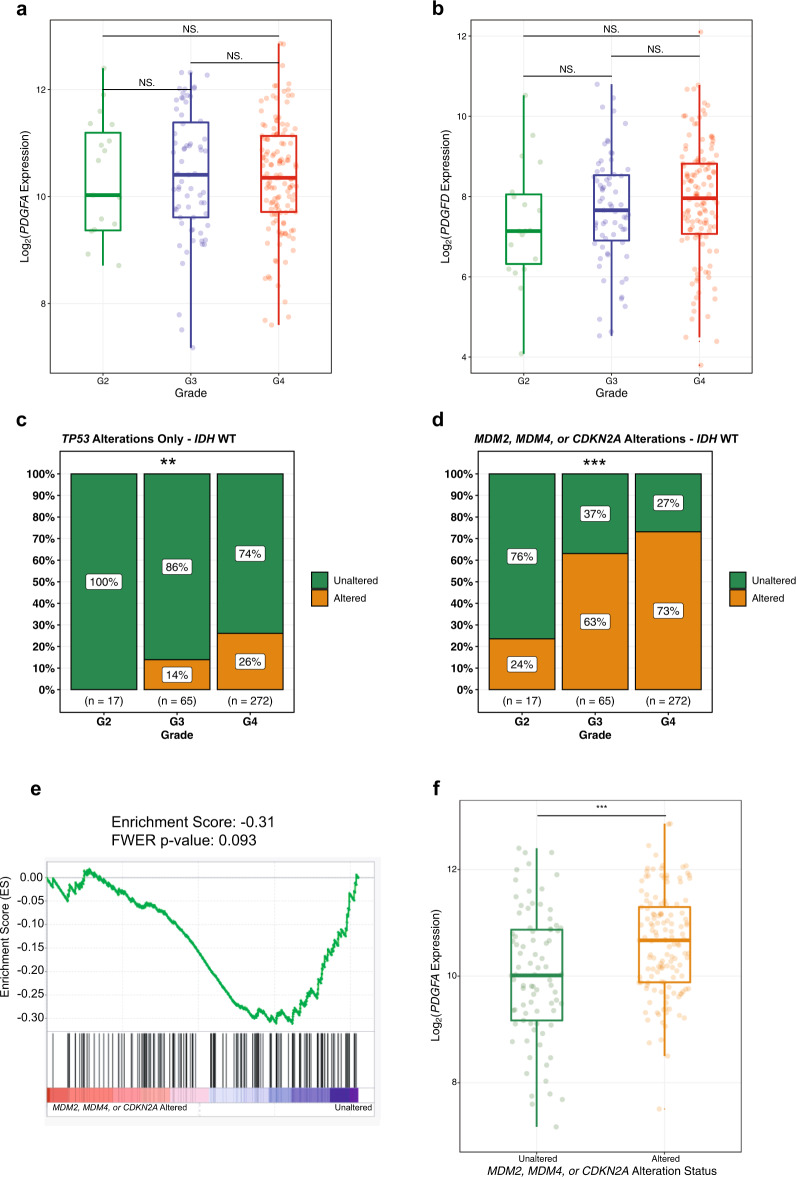
The evolution of *PDGFA*/*PDGFD* overexpressing *IDH* WT LGAs to higher-grade disease is accompanied by a progressive increase in p53 pathway mutations. **a**, **b** Box plots depicting the quantification of *PDGFA* and *PDGFD* expression by grade in *IDH* WT astrocytic gliomas. **c**, **d** Bar plots showing the proportion of *IDH* WT astrocytic gliomas harboring alterations by grade in the *TP53* gene and any of the following genes: *MDM2*, *MDM4*, or *CDKN2A*. **e** Gene set enrichment analysis plot, enrichment score (ES) and family-wise error rate (FWER) *p* value showing the depletion of a p53 target gene set in *MDM2*/*MDM4*/*CDKN2A* altered *IDH* WT LGAs. **f** Box plots depicting the quantification of *PDGFA* expression in *MDM2*/*MDM4*/*CDKN2A* altered *versus* unaltered among *IDH* WT LGAs. (***P < 0.001). In Box plots, the lower bound, center line and upper bound correspond to the first, second and third quartiles, respectively, and whiskers correspond to the maximum and minimum data values.

To further explore the more aggressive behavior of *IDH* WT LGAs and to better understand how they evolve to higher grades, we evaluated the expression of *PDGFA* and *PDGFD* genes in WHO grade II, and grade III tumors and in GBMs. We found that a high level of expression of *PDGFA* and *PDGFD* was a constant feature of the *IDH* WT disease, irrespective of histological grade (*n* = 219, Fig. 4a, b). These observations reveal that overexpression of *PDGFA* is an early feature of *IDH* WT LGAs that persists as grade 2 tumors evolve to grade 3 lesions and on to GBM.

We then assessed the mutational status of the p53 pathway, because loss or inactivation of *TP53* has been hypothesized to cooperate with PDGF signaling to promote *IDH* WT GBM^9^, and because *TP53* compromise (i.e., null or heterozygous) is a prerequisite for PDGFA-mediated in vitro transformation of neural progenitor cells^10^. First, we assessed single nucleotide variants (SNVs) in *TP53*. Unlike *IDH* mutant LGAs, which had a high proportion of *TP53* SNVs (Supplementary Fig. S7a) in tumors of all WHO grades (*P* = 0.75, *n* = 231), SNVs were not found in grade II WT LGAs and were only detected in some grade 3 tumors and GBMs (*P* = 0.0078, *n* = 354, Fig. 4c). Since alterations of the p53 pathway can occur in ways other than point-mutation, we assessed copy number variants (CNVs) of *CDKN2A*, which encodes the positive regulator of p53, p14ARF, and variants of the negative regulators of p53, *MDM2* and *MDM4*^21^. We noted a progressive increase in the frequency of *CDKN2A* and *MDM2*/*MDM4* alterations with increasing grade in WT tumors (*P* = 5.3 * 10^−5^, *n* = 354, Fig. 4d). Moreover, pathway disruption accompanied progression to GBM from a lower grade WT tumor in virtually all cases (*P* = 5.4 * 10^−11^, *n* = 354; Supplementary Fig. S7b).

Lastly, we sought confirmation that deletion of *CDKN2A* and amplification of *MDM2 or MDM4* deregulated the p53 pathway in *IDH* WT LGAs. Gene set enrichment analysis (GSEA) identified a negative association between a specified list of *TP53* target genes and an alteration of *CDKN2A/MDM2*/*MDM4* in *IDH* WT LGAs (Fig. 4e). *IDH* WT tumors with alterations in *CDKN2A/MDM2*/*MDM4* cluster had elevated expression of *PDGFA versus* LGAs in which at least one of *CDKN2A, MDM2, or MDM4* was unaltered (*P* = 0.00017, *n* = 222, Fig. 4f). These data imply that a determinant of the progression of WT grade 2 LGAs, to grade 3 LGAs, and beyond to GBMs, may be inactivation of the p53 pathway by one of several mechanisms.

## Discussion

The biology that underlies the contrasting clinical features of *IDH* WT and *IDH* mutant LGAs is poorly understood. Here, we report differences in the expression of *PDGF* gene-family members, particularly *PDGFA*; differences in expression of biomarkers of invasiveness, immune evasion, and genomic instability; and differences in the type and temporality of p53 pathway alterations that suppress function. Each of these features significantly associates with *IDH* mutational status and may contribute to the aggressive behavior and short survival of patients with WT tumors, on the one hand, and to the indolent nature and long survival of those with mutant tumors on the other^4^. However, with regard to the behavior of the *IDH* WT cases, readers are cautioned that the databases upon which this study is based were assembled before grade 2 and grade 3 *IDH* WT diffuse fibrillary astrocytic gliomas with TERT promoter mutations, EGFR amplification, and/or a combination of gain of complete chromosome 7 and loss of complete chromosome 10 (+7/−10) were renamed GBMs. Hence, some of the lower grade tumors in this analysis would now be listed as GBMs. A possible effect of this shift in classification on our survival analyses is acknowledged.

Two findings that emerge from this analysis warrant further comment. First, *PDGFA* was highly differentially expressed between *IDH* WT and mutant *IDH* LGAs. Overexpression in WT cases is consistent with the report of Ozawa et al.^9^ in which overexpression of *PDGFA* was predicted to be an early alteration in the pathogenesis of human non-GCIMP (i.e., *IDH* WT) GBM, and when overexpressed in p53 null mice, led to the generation of GBM-like tumors. Moreover, in our hands, continuous exposure to PDGFA induced the malignant transformation of cultured p53 null and heterozygous murine neural progenitor cells isolated from the Sub-Ventricular Zone (SVZ) of young adult mice^10^. Transformation in this setting was characterized by gains and losses of whole chromosomes and arms of chromosomes in neural progenitor cells and by their evolution to a PDGFA-independent proliferative phenotype with the capacity to generate infiltrating GBM-like cancers in the brains of immune-competent mice. Furthermore, amplification of a segment of chromosome 7 containing the *PDGFA* locus, which was only seen in *IDH* WT LGAs, emerged as a putative mechanism for the high expression of *PDGFA* and was associated with worse prognosis. Also consistent with worse patient outcomes was the finding that overexpression of *PDGFA* in *IDH* WT LGAs was associated with high aneuploidy scores and markers of immune evasion.

In contrast, *PDGFA* was expressed at low levels in mutant cases in association with promoter methylation. Low expression was also associated with lesser degrees of genomic instability and lower levels of expression of genes linked to immune evasion, characteristics that might contribute to slower rates of malignant progression and a more favorable prognosis. In addition, we observed that the *PDGFRA* receptor was overexpressed in mutant tumors, perhaps as a compensatory response to downregulation of its key ligand, *PDGFA*. Such dramatic differences in the expression of ligands and receptors from the same growth factor family suggest that genes in this pathway play important but different roles in the pathogenesis of mutant and WT LGAs.

A second insight that emerged from this analysis pertains to perturbations of the p53 pathway in LGA. Although p53 alterations were essentially universal among the LGAs evaluated here, the nature and ‘staging’ of these alterations differed between *IDH* WT and *IDH* mutant LGAs in a way that may bear on their different clinical behaviors. As noted previously, point mutations of *TP53* (i.e., SNVs), primarily located in the DNA binding domain of the gene, were an early and constant feature of *IDH* mutant LGAs. They were found in virtually all *IDH* mutant grade II, grade III, and high-grade (i.e., grade IV) lesions. In contrast, *TP53* SNVs were not detected in *IDH* WT grade 2 LGAs and were less commonly observed than other types of p53 pathway alterations in grade 3 WT tumors and in GBMs. Instead, in *IDH* WT LGAs the p53 pathway was inactivated by a variety of different mechanisms including deletion of the p53-positive regulator *CDKN2A* and overexpression of the p53-negative regulators, *MDM2* and *MDM4*. These qualitative differences in p53 alterations between *IDH* WT and mutant LGAs have not been highlighted previously.

These analyses illustrate the scope of genomic reprogramming that occurs in the diffuse astrocytic gliomas in association with the presence or absence of an *IDH* mutation and signal potentially important roles of the PDGF and p53 pathways in mediating their different behaviors. Finally, these data generate hypotheses that can be explored in models of LGA and GBM.

## Methods

### Data analysis and statistical tests

Data processing and analyses were performed on R version 4.0.0. All statistical tests were two-sided.

### Datasets used

TCGA clinical data for LGG and GBM cases was downloaded from Supplementary Table 1 in Ceccarelli et al.^22^. This dataset was utilized for annotated information on the grade, *IDH* status, and 1p/19q codeletion status of TCGA gliomas. *IDH* status included mutations of both the *IDH1* and *IDH2* genes^22^. Pan-cancer (including TCGA-GBM/LGG) survival data was downloaded from Supplementary Table 1 in Liu et al.^23^. P53 pathway genes (*TP53, MDM2, MDM4, and CDKN2A*) were queried for mutations and CNVs in the TCGA-GBMLGG dataset on the cBio Cancer Genomics Portal (cbioportal.org)^24^. The corresponding raw dataset for the OncoPrint generated by cBioPortal was downloaded for analysis on a third-party platform. Aneuploidy Scores (AS) for TCGA-GBM/LGG samples were acquired from Supplementary Table 2 provided in the study by Taylor et al.^17^ Three hundred forty-four out of 347 LGA cases had mutation, methylation and transcriptome data available for analysis. Of note, these datasets were built before the new WHO nomenclature for central nervous system tumors were published^25^ and *IDH* WT diffuse astrocytomas with *TERT* promoter mutations, EGFR amplification, and/or +7/−10 were renamed GBM.

### Gene expression, copy number, and methylation datasets

Normalized level 3 RSEM RNA-seq data, segmented copy number data from SNP6 arrays, and Infinium 450k methylation array data for TCGA-GBMLGG samples was downloaded from the Broad GDAC Firehose (https://gdac.broadinstitute.org). For copy number analysis, probes were filtered to those overlapping the region containing the *PDGFA* gene (chromosome 7 between 536897 bp and 559481 bp) or the *PDGFD* gene (chromosome 11 between 103777914 bp and 104035027 bp). Absolute copy number values were computed by transforming segment means (absolute copy # = 2 × 2^(segment mean)^). Methylation probes cg15454385 and cg03145963 were used as the representative probes to study *PDGFA* and *PDGFD* promoter methylation status, respectively.

### Filtering of gliomas, and classification of LGAs

Depending on the analysis requirements, filters were utilized to select glioma samples based on their grade or molecular alteration status (*IDH* status, 1p/19q codeletion, or p53 pathway alterations). For the purposes of this analysis, LGAs were defined as WHO grade II and grade III gliomas without 1p/19 codeletions.

### Differential expression analysis of IDH WT LGAs vs. IDH mutant LGAs in TCGA

Level 3 RNA-Seq by Expectation-Maximization (RSEM) data was downloaded for TCGA-GBM/LGG samples from GDAC Firehose (https://gdac.broadinstitute.org). Samples were filtered for tumors and subsequently for LGAs, as per the criteria described above. Samples classified as NA for *IDH* status in the clinical dataset were not considered further. Differential expression analysis was performed between *IDH* WT and *IDH* mutant LGAs using the DESeq2 package^26^. A differentially expressed gene (DEG) list was generated with an adjusted *P* value threshold of 0.001 and log_2_(fold change) threshold of +1. *P* value adjustment was performed with the application of the Benjamini-Hochberg method.

### Pathway enrichment analysis of DEGs between IDH WT and IDH mutant LGAs

Pathway enrichment analysis was performed on a smaller DEG list (with an adjusted *P* value threshold of 0.001 and log_2_(fold change) threshold of +2) using the ReactomePA package^27^. As per the differential expression analysis, *P* value adjustment was performed using the Benjamini-Hochberg method. Since there were multiple changes related to collagen, scavenger receptors, and acetylcholine receptors, these alterations were collapsed into one pathway hit each.

### Gene set enrichment analyses

p53 pathway genes were identified from the Molecular Signature Database (MSigDB v6.2, C2 collection; https://www.gsea-msigdb.org/gsea/msigdb/genesets.jsp? collection = C2). The list of TGF-β upregulated genes and cancer-associated ECM (C-ECM) genes were downloaded from the supplementary material in the study by Chakravarthy et al.^20^. These genesets were used to compute single sample gene enrichment analysis (ssGSEA) scores using the gene set variation analysis (GSVA) R package^28,29^. For a pre-defined gene set, ssGSEA calculates an enrichment score based on enriched and depleted gene expression for each case. Gene set enrichment analysis (GSEA) was performed with the Broad GSEA 4.0.1 software. GSEA permutation type was set to “phenotype” and 1000 permutations were performed.

### Survival analyses and Kaplan–Meier visualizations

Cox proportional hazards models were fit on R with the survival package. Prior to visualization, all survival associations were confirmed to be significant in univariate Cox proportional hazards models with the continuous variables as covariates. Differences in surviving fractions between groups were visualized via Kaplan–Meier curves generated using the survminer R package. Cut-points for continuous variables were identified by the method reported by Contal and O’Quigley^30^.

### Validation cohorts

Gene expression and clinical data from two additional glioma datasets were obtained (GSE16011 and REMBRANDT). For both datasets, gliomas were filtered to contain only LGAs (i.e., WHO Grade II or Grade III tumors that were non-1p/19q co-deleted).

### Statistical visualizations

Graphs with statistical information and bar plots were generated using the ggstatsplot R package.

## Supplementary information

Supplementary Figues and Legends

## Data Availability

TCGA clinical data for LGG and GBM cases was downloaded from Supplementary Table 1 in Ceccarelli et al. Pan-cancer (including TCGA-GBM/LGG) survival data was downloaded from Supplementary Table 1 in Liu et al. p53 pathway genes (TP53, MDM2, MDM4, and CDKN2A) were queried for mutations and CNVs in the TCGA-GBMLGG dataset on the cBio Cancer Genomics Portal (cbioportal.org). The corresponding raw dataset for the OncoPrint generated by cBioPortal was downloaded for analysis on a third-party platform. Aneuploidy Scores (AS) for TCGA-GBM/LGG samples were acquired from Supplementary Table 2 provided in the study by Taylor et al. Normalized level 3 RSEM RNA-seq data, segmented copy number data from SNP6 arrays, and Infinium 450k methylation array data for TCGA-GBMLGG samples was downloaded from the Broad GDAC Firehose (https://gdac.broadinstitute.org). RNA-Seq by Expectation-Maximization (RSEM) data was downloaded for TCGA-GBM/LGG samples from GDAC Firehose (https://gdac.broadinstitute.org). p53 pathway genes were identified from the Molecular Signature Database (MSigDB v6.2, C2 collection; https://www.gsea-msigdb.org/gsea/msigdb/genesets.jsp? collection = C2). The list of TGF-β upregulated genes and cancer-associated ECM (C-ECM) genes were downloaded from the supplementary material in the study by Chakravarthy et al. Gene expression and clinical data from two additional glioma datasets were obtained (GSE16011 and REMBRANDT). For both datasets, gliomas were filtered to contain only LGAs (i.e., WHO Grade II or Grade III tumors that were non-1p/19q co-deleted).
