# Supplementary material for: PDGF gene expression and p53 alterations contribute to the biology of diffuse astrocytic gliomas
Source: NPJ Genom Med. 2023 Feb 25;8:6. doi: 10.1038/s41525-023-00351-2 (PMC9968280; doi:10.1038/s41525-023-00351-2)
Supplement: Supplementary file 1 — Supplementary Figues and Legends [file 41525_2023_351_MOESM1_ESM.pdf]

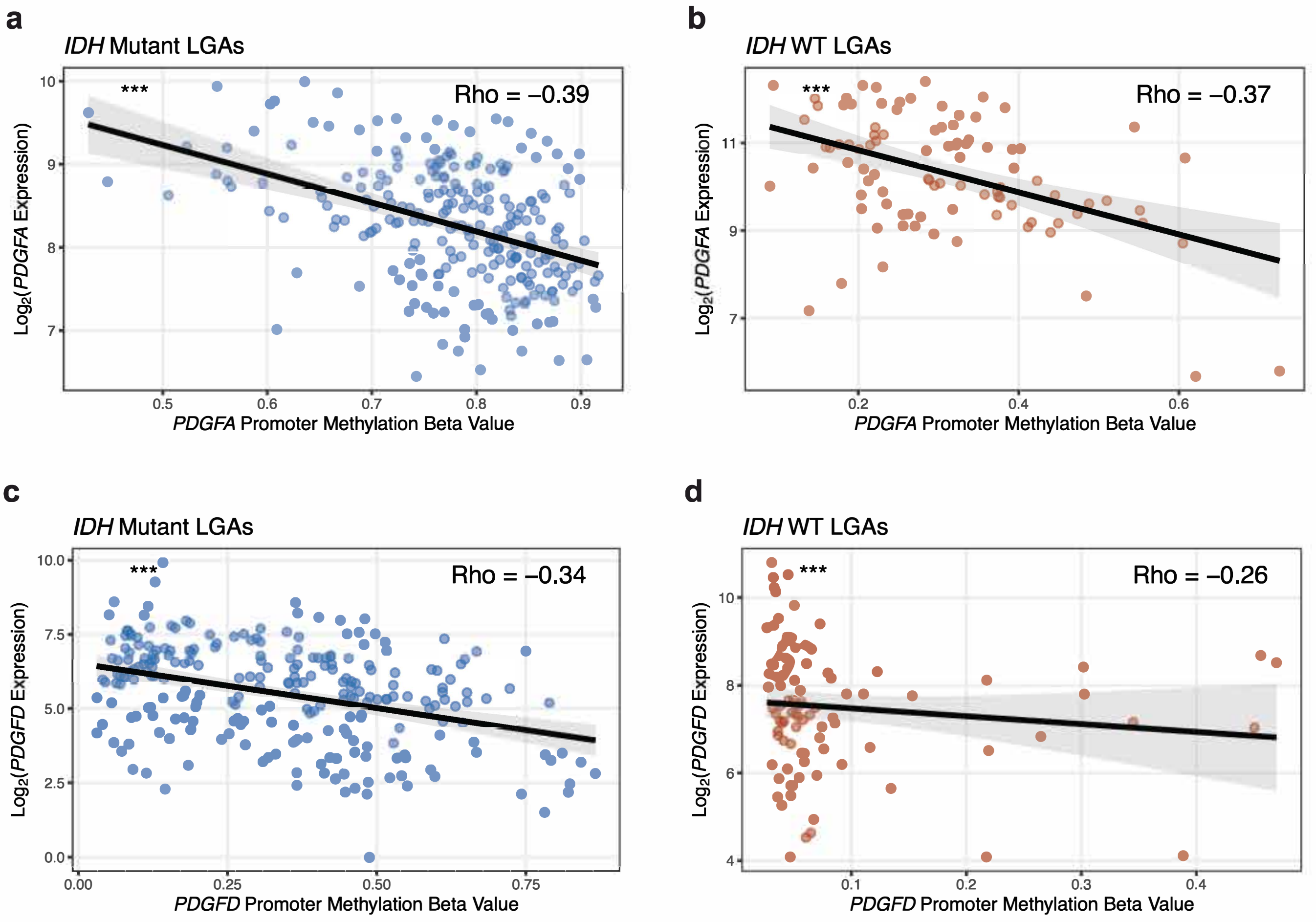

Supplementary Figure 1: PDGFA and PDGFD expression are negatively regulated by promoter methylation. Scatterplots showing the negative correlation between PDGFA promoter methylation and PDGFA expression in a. IDH mutant and b. IDH WT LGAs. Scatterplots showing the negative correlation between PDGFD promoter methylation and PDGFD expression in c. IDH mutant and d. IDH WT LGAs. Spearman's Rho values are reported. (* $P < 0.05$, *** $P < 0.001$).

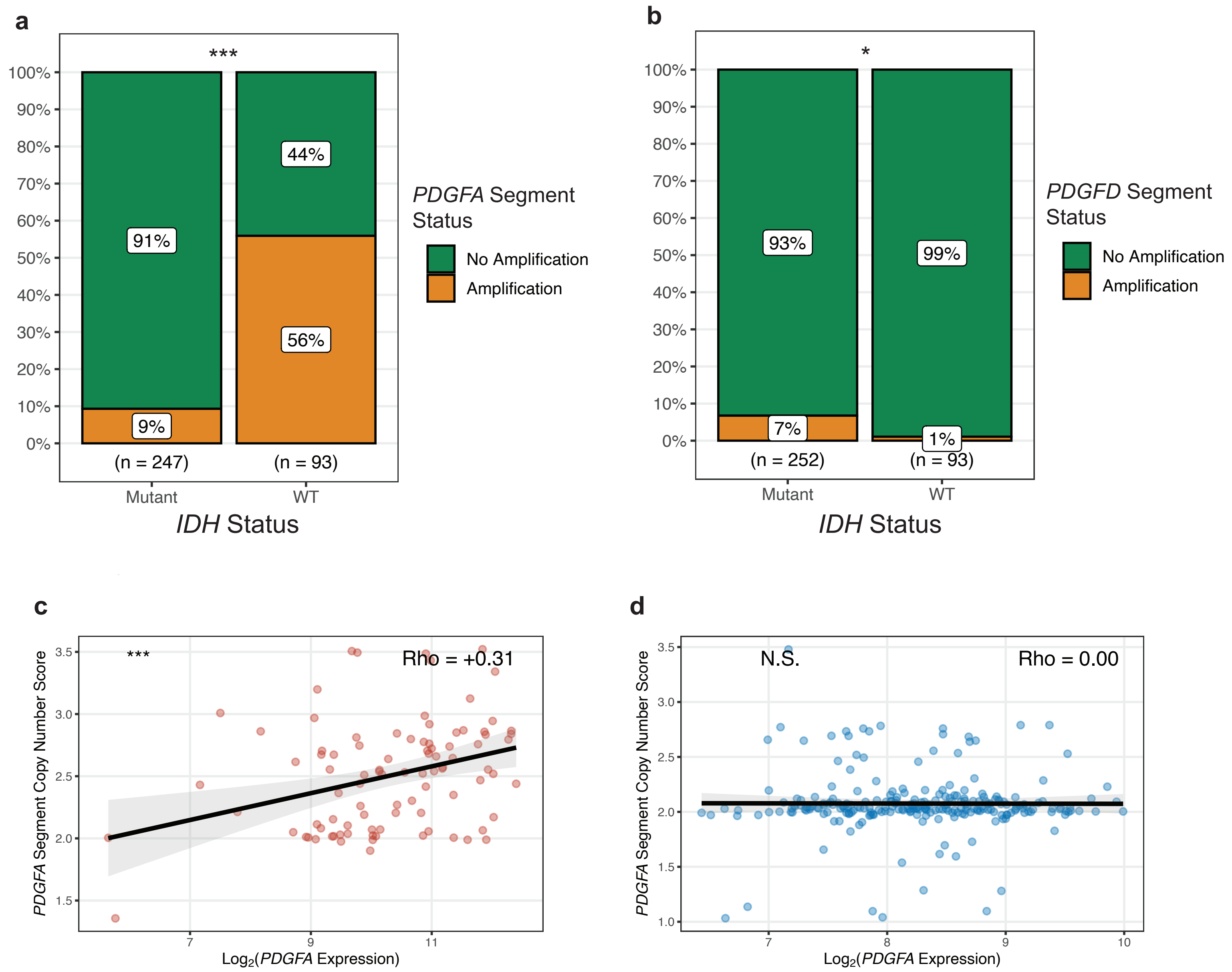

Supplementary Figure 2. *PDGFA* but not *PDGFD* expression is regulated by chromosomal amplification. Bar plots showing the proportions of samples with **a.** *PDGFA* and **b.** *PDGFD* segment amplification in *IDH* mutant and *IDH* WT LGAs. Scatterplots showing the correlation between the absolute copy number of the chromosomal segment containing *PDGFA* and *PDGFA* expression in **c.** *IDH* WT and **d.** *IDH* mutant LGAs. (* $P < 0.05$, *** $P < 0.001$; N.S.: not significant, $P > 0.1$).

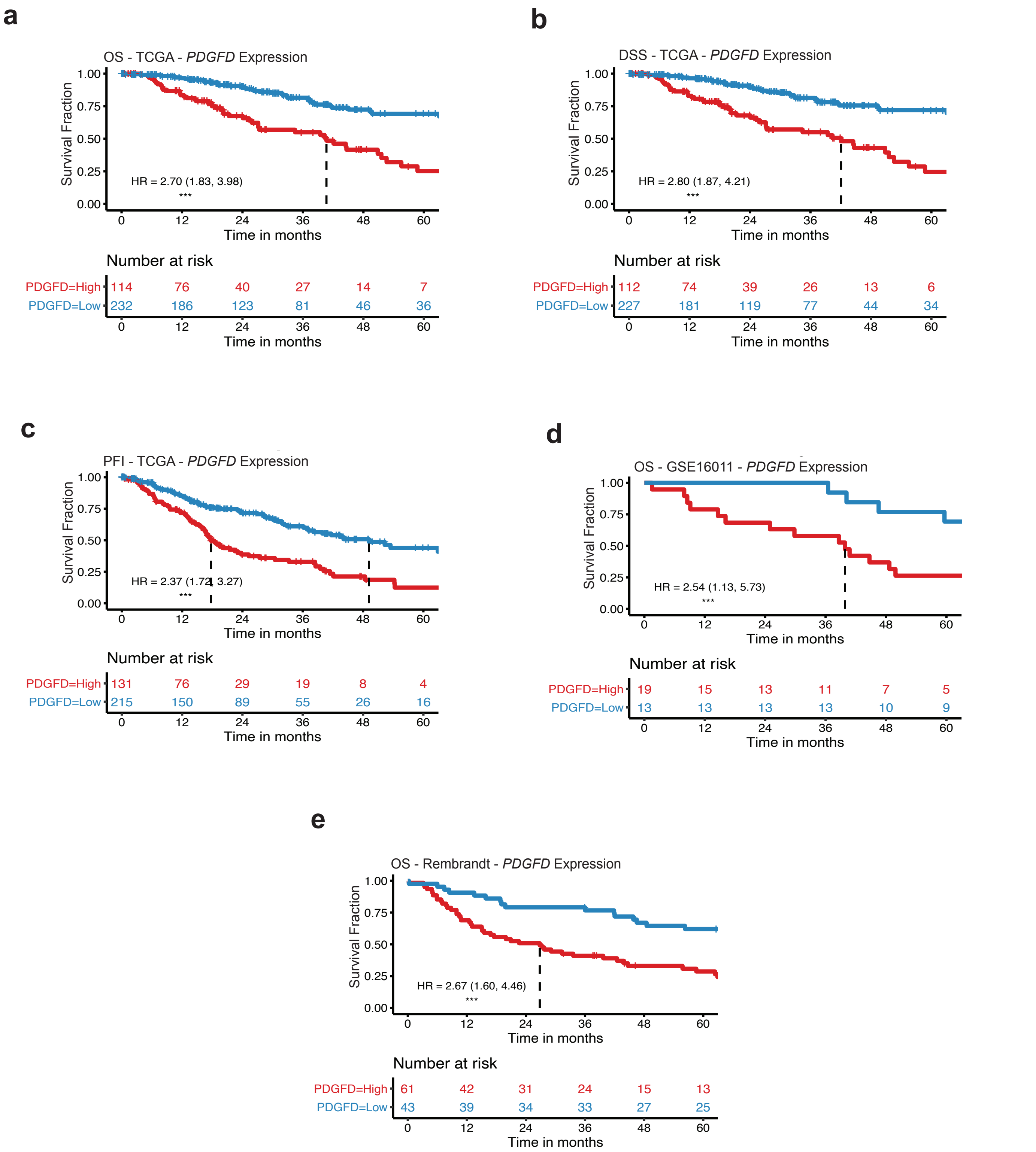

Supplementary Figure 3. *PDGFD* expression is associated with significantly worse prognosis in LGA. KM survival curves for **a.** OS **b.** DSS and **c.** PFI showing the separation of TCGA LGA patients into risk groups based on *PDGFD* expression. K M survival curves validating the association between *PDGFD* expression and OS in LGA patients from the **d.** GSE16011 and **e** REMBRANDT datasets. Hazard ratios (HR) and their respective 95% confidence intervals from univariate Cox proportional hazards analysis of the dichotomized expression groups are reported for each KM curve. (* $P < 0.05$, *** $P < 0.001$).

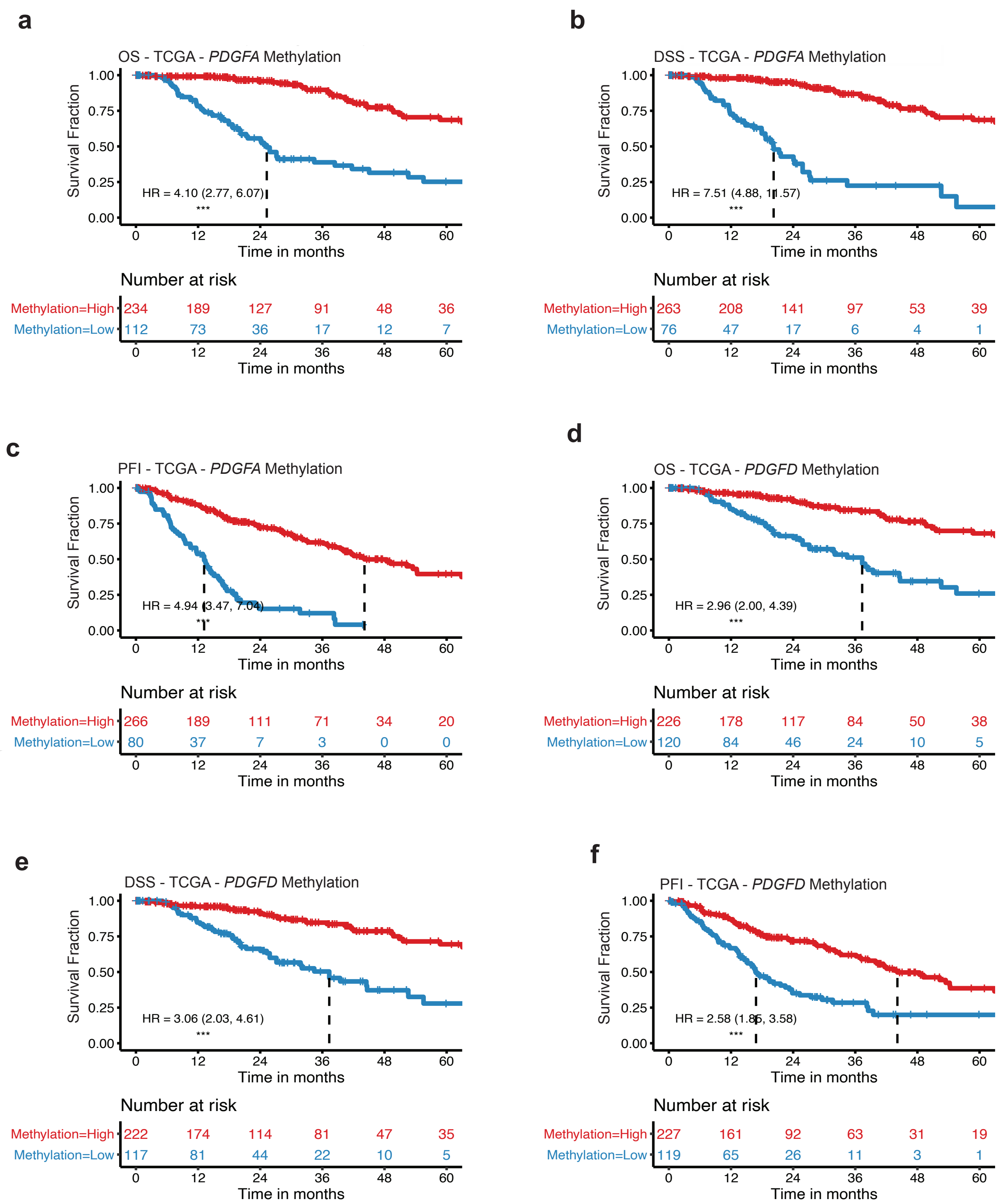

Supplementary Figure 4. Increased *PDGFA* and *PDGFD* promoter methylation are associated with improved prognosis in LGA. KM survival curves for **a.** OS **b** DSS and **c.** PFI showing the separation of TCGA LGA patients into risk groups based on *PDGFA* promoter methylation. KM survival curves for **d.** OS **e.** DSS and **f.** PFI showing the separation of TCGA LGA patients into risk groups based on *PDGFD* promoter methylation. Hazard ratios (HR) and their respective 95% confidence intervals from univariate Cox proportional hazards analysis of the dichotomized expression groups are reported for each KM curve. (*** $P < 0.001$).

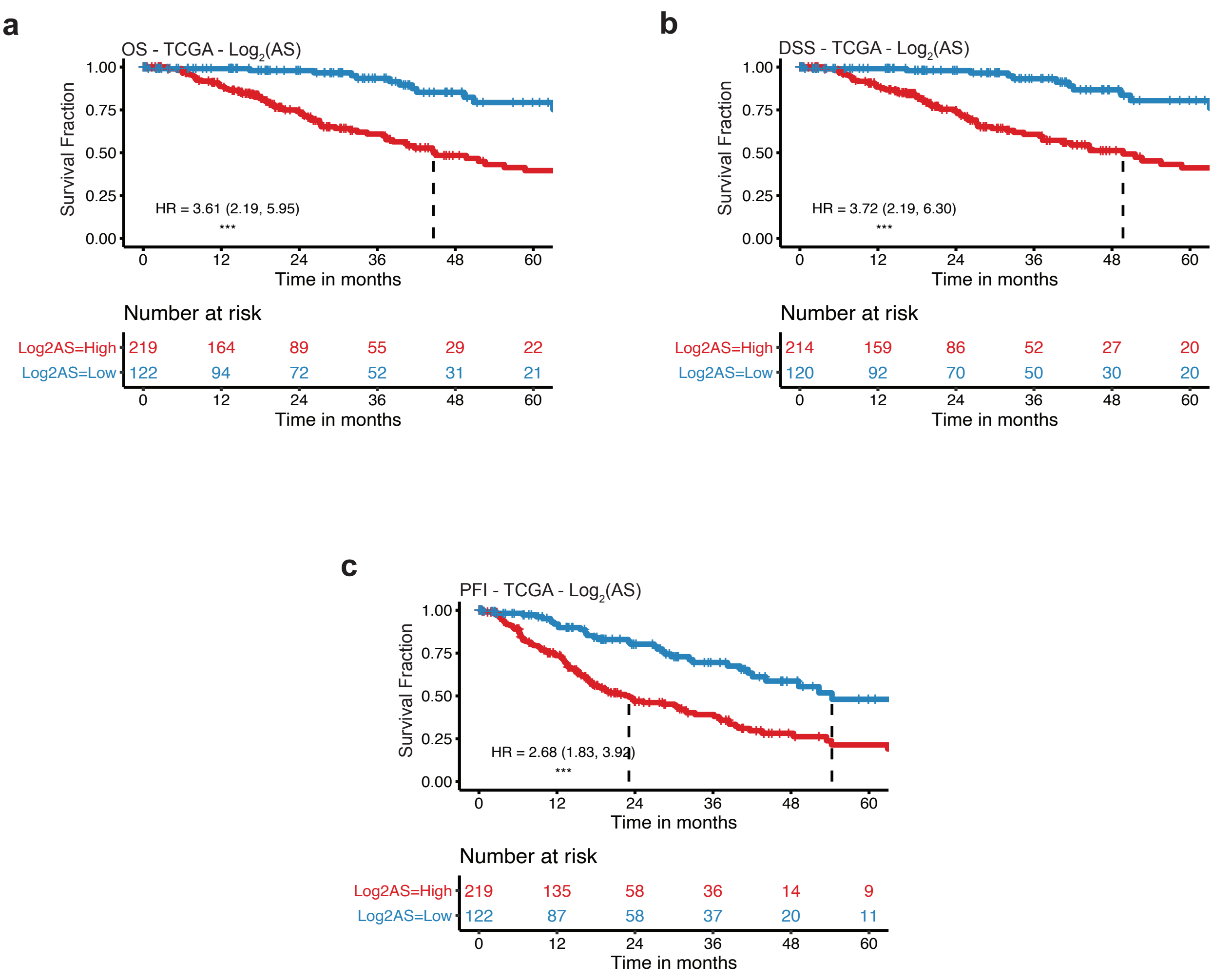

Supplementary Figure 5. Increased aneuploidy is associated with significantly worse prognosis in LGA. KM survival curves for **a.** OS **b.** DSS and **c.** PFI showing the separation of TCGA LGApatients into risk groups based on AS. Hazard ratios (HR) and their respective 95% confidence intervals from univariate Cox proportional hazard analysis of the dichotomized groups are reported for each KM curve. (*** $P < 0.001$).

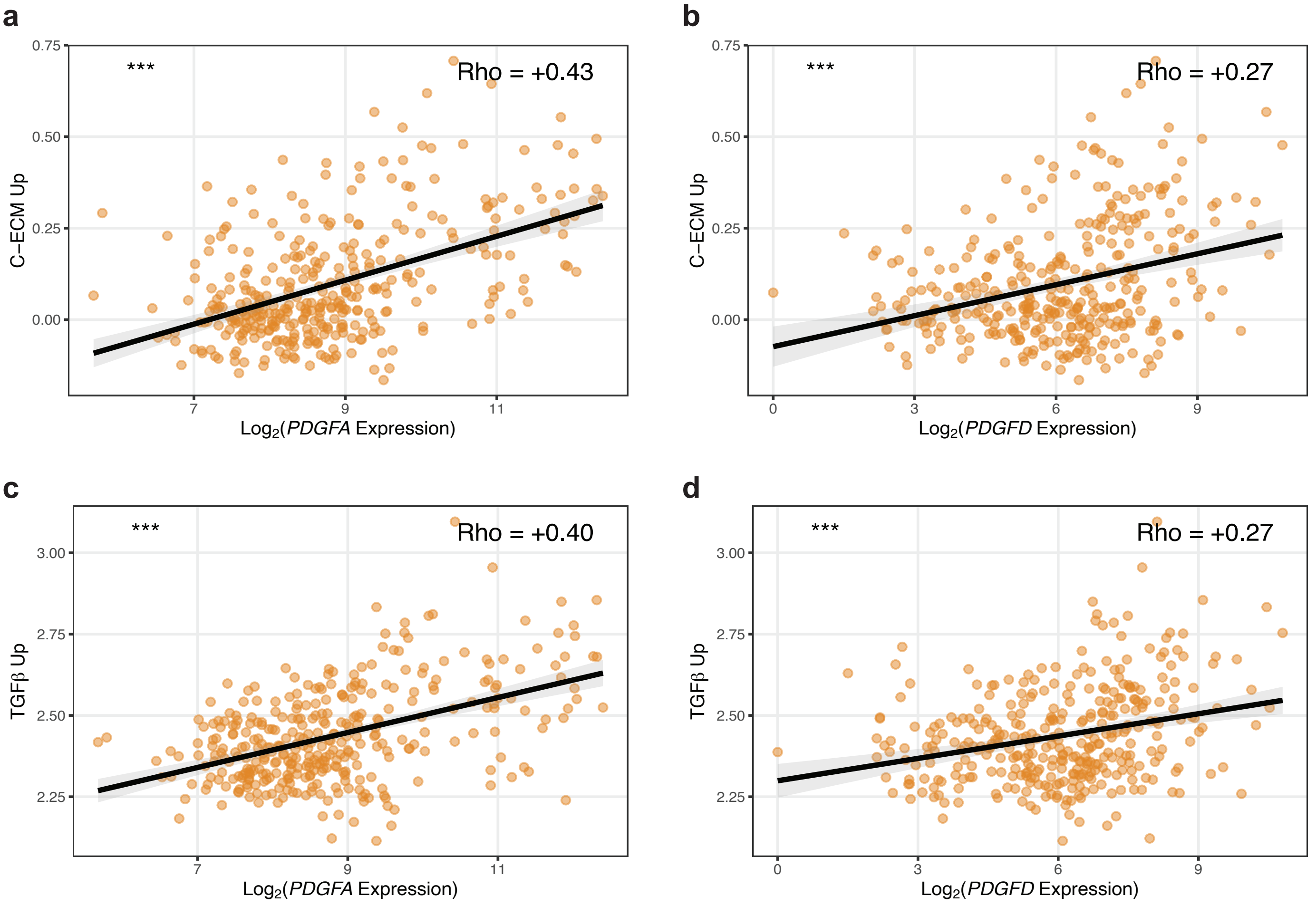

Supplementary Figure 6. *PDGFA* and *PDGFD* expression are associated with markers of immunosuppression. Scatterplots showing the positive correlation of ssGSEA scores of C-ECM upregulated genes with **a** *PDGFA* and **b** *PDGFD* expression. Scatterplots showing the positive correlation of ssGSEA scores of TGF-β upregulated target genes with **c** *PDGFA* and **d** *PDGFD* expression. Spearman's Rho values are reported. (* $P < 0.05$, *** $P < 0.001$; N.S.: not significant, $P > 0.1$).

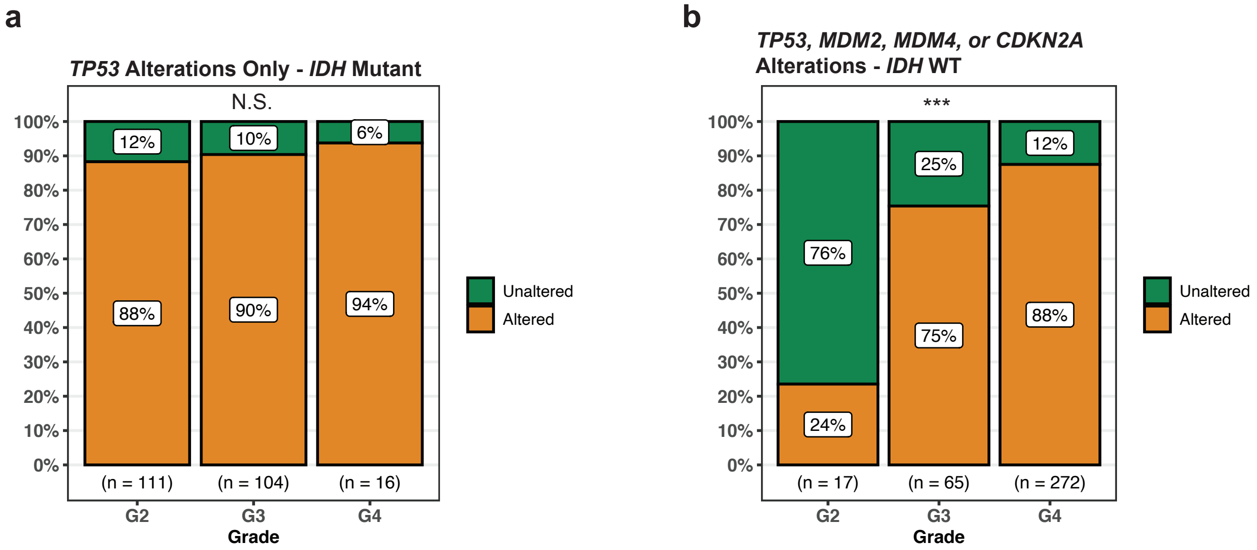

Supplementary Figure 7: IDH WT glioma progression from low to high grade is marked by an increase in the frequency of p53 pathway alterations. a Bar plot showing the proportion of TP53 alterations by grade in IDH mutant astrocytic gliomas. b Bar plot showing the proportion of TP53, MDM2, MDM4 and CDKN2A alterations by grade in IDH WT astrocytic gliomas. (*** $P < 0.001$; N.S.: not significant, $P > 0.1$).
